# The Inferelator: an algorithm for learning parsimonious regulatory networks from systems-biology data sets *de novo*

**DOI:** 10.1186/gb-2006-7-5-r36

**Published:** 2006-05-10

**Authors:** Richard Bonneau, David J Reiss, Paul Shannon, Marc Facciotti, Leroy Hood, Nitin S Baliga, Vesteinn Thorsson

**Affiliations:** 1 New York University, Biology Department, Center for Comparative Functional Genomics, New York, NY 10003, USA; 2 Courant Institute, NYU Department of Computer Science, New York, NY 10003, USA; 3 Institute for Systems Biology, Seattle, WA 98103-8904, USA

## Abstract

We present a method (the Inferelator) for deriving genome-wide transcriptional regulatory interactions, and apply the method to predict a large portion of the regulatory network of the archaeon *Halobacterium NRC-1*. The Inferelator uses regression and variable selection to identify transcriptional influences on genes based on the integration of genome annotation and expression data. The learned network successfully predicted *Halobacterium*'s global expression under novel perturbations with predictive power similar to that seen over training data. Several specific regulatory predictions were experimentally tested and verified.

## Abstract 2

The Inferelator, a method for deriving genome-wide transcriptional regulatory interactions, successfully predicted global expression in *Halobacterium *under novel perturbations.

## Background

Distilling regulatory networks from large genomic, proteomic and expression data sets is one of the most important mathematical problems in biology today. The development of accurate models of global regulatory networks is key to our understanding of a cell's dynamic behavior and its response to internal and external stimuli. Methods for inferring and modeling regulatory networks must strike a balance between model complexity (a model must be sufficiently complex to describe the system accurately) and the limitations of the available data (in spite of dramatic advances in our ability to measure mRNA and protein levels in cells, nearly all biologic systems are under-determined with respect to the problem of regulatory network inference).

A major challenge is to distill, from large genome-wide data sets, a reduced set of factors describing the behavior of the system. The number of potential regulators, restricted here to transcription factors (TFs) and environmental factors, is often on the same order as the number of observations in current genome-wide expression data sets. Statistical methods offer the ability to enforce parsimonious selection of the most influential potential predictors of each gene's state. A further challenge in regulatory network modeling is the complexity of accounting for TF interactions and the interactions of TFs with environmental factors (for example, it is known that many transcription regulators form heterodimers, or are structurally altered by an environmental stimulus such as light, thereby altering their regulatory influence on certain genes). A third challenge and practical consideration in network inference is that biology data sets are often heterogeneous mixes of equilibrium and kinetic (time series) measurements; both types of measurements can provide important supporting evidence for a given regulatory model if they are analyzed simultaneously. Last, but not least, is the challenge resulting from the fact that data-derived network models be predictive and not just descriptive; can one predict the system-wide response in differing genetic backgrounds, or when the system is confronted with novel stimulatory factors or novel combinations of perturbations?

A significant body of work has been devoted to the modeling and learning of regulatory networks [1-3]. In these studies regulatory interactions and dynamics are modeled with varying degrees of detail and model flexibility and, accordingly, such models can be separated into general classes based on the level of detail with which they model individual regulatory interactions [1,2]. At the highest level of detail lie differential equations and stochastic models, which provide detailed descriptions of regulatory systems and can be used to simulate systems dynamics, but they are computationally demanding and require accurate measurement of a large number of parameters. Hence, these simulations have primarily been carried out for small-scale systems (relative to the full, genome-wide, regulatory circuit for a given organism); often these studies model systems that have been studied in great detail for decades, such as the galactose utilization pathway in yeast and the early development of sea urchin. At the other end of the model complexity spectrum lie Boolean networks [4], which assume that genes are simply on or off, and include standard logic interactions (AND, OR, XOR, and so on). Despite this simplification of regulatory dynamics and interactions, these approaches have the advantages of simplicity, robustness (they can be learned with significantly fewer data), and ease of interpretation [5]. Recent probabilistic approaches to modeling regulatory network on the genome-wide scale use Bayesian networks to model regulatory structure, *de novo*, at the Boolean level [6-11].

Additive linear or generalized linear models take an intermediate approach, in terms of model complexity and robustness [12-15]. Such models describe each gene's expression level as a weighted sum of the levels of its putative predictors. Inclusion of functions that modify the linear response produced by these additive methods (sometimes referred to as squashing functions) allows some biologically relevant nonlinear processes (for example, promoter saturation) to be modeled. An advantage of linear and generalized linear models is that they draw upon well developed techniques from the field of statistical learning for choosing among several possible models and efficiently fitting the parameters of those models.

Learning and/or modeling of regulatory networks can be greatly aided by reducing the dimensionality of the search space before network inference. Two ways to approach this are limiting the number of regulators under consideration and grouping genes that are co-regulated into clusters. In the former case, candidates can be prioritized based on their functional role (for example, limiting the set of potential predictors to include only TFs, and grouping together regulators that are in some way similar). In the latter case, gene expression clustering, or unsupervised learning of gene expression classes, is commonly applied. It is often incorrectly assumed that co-expressed genes correspond to co-regulated genes. However, for the purposes of learning regulatory networks it is desirable to cluster genes on the basis of co-regulation (shared transcriptional control) as opposed to simple co-expression. Furthermore, standard clustering procedures assume that co-regulated genes are co-expressed across all observed experimental conditions. Because genes are often regulated differently under different conditions, this assumption is likely to break down as the quantity and variety of data grow.

Biclustering was developed to address better the full complexity of finding co-regulated genes under multifactor control by grouping genes on the basis of coherence under subsets of observed conditions [10,16-22]. We developed an integrated biclustering algorithm, named cMonkey (Reiss DJ, Baliga NS, Bonneau R, unpublished data), which groups genes and conditions into biclusters on the basis of the following: coherence in expression data across subsets of experimental conditions; co-occurrence of putative *cis*-acting regulatory motifs in the regulatory regions of bicluster members; and the presence of highly connected subgraphs in metabolic [23] and functional association networks [24-26]. Because cMonkey was designed with the goal of identifying putatively co-regulated gene groupings, we use it to 'pre-cluster' genes before learning regulatory influences in the present study. cMonkey identifies relevant conditions in which the genes within a given bicluster are expected to be co-regulated, and the inferred regulatory influences on the genes in each bicluster pertain to (and are fit using) only those conditions within each bicluster. In principle, the algorithm described in this work can be coupled with other biclustering and clustering algorithms.

Here we describe an algorithm, the Inferelator, that infers regulatory influences for genes and/or gene clusters from mRNA and/or protein expression levels. The method uses standard regression and model shrinkage (L1 shrinkage) techniques to select parsimonious, predictive models for the expression of a gene or cluster of genes as a function of the levels of TFs, environmental influences, and interactions between these factors [27]. The procedure can simultaneously model equilibrium and time course expression levels, such that both kinetic and equilibrium expression levels may be predicted by the resulting models. Through the explicit inclusion of time and gene knockout information, the method is capable of learning causal relationships. It also includes a novel solution to the problem of encoding interactions between predictors into the regression. We discuss the results from an initial run of this method on a set of microarray observations from the halophilic archaeon *Halobacterium NRC-1*.

## Results and discussion

### The inferred global regulatory network for *Halobacterium* NRC-1

We applied our method to the Halophilic archaeon *Halobacterium NRC-1*. The *Halobacterium *genome contains 2,404 nonredundant genes, of which 124 are annotated to be known or putative TFs [28,29]. The biclustering and network inference procedure were performed on a recently generated data set containing 268 mRNA microarray measurements of this archaeon under a wide range of genetic and environmental perturbations ('Kaur A, Pan M, Meislin M, El-Geweley R, Baliga NS' and 'Whitehead K, Kish A, Pan M, Kaur A, King N, Hohmann L, Diruggiero J, Baliga NS', personal communications), [30,31]. Several TFs do not change significantly in their expression levels in the data set; of the 124 identified TFs, 100 exhibited a significant change in expression levels across the data set, and the remaining 24 TFs were excluded from the set of potential influences (see Materials and methods, below) [32]. Strongly correlated TFs (those with correlation greater than 0.85) were further grouped, yielding 72 regulators (some representing multiple correlated regulators). To these 72 potential regulators were added 10 environmental factors for a total of 82 possible predictors for the 1,934 genes with significant signal in the data set. In addition to this main data set, 24 new experiments (collected after model fitting) were used for independent error estimation subsequent to the network inference procedure.

The cMonkey method (Reiss DJ, Baliga NS, Bonneau R, unpublished data) was applied to this data set (original 268 conditions) to bicluster genes and conditions, on the basis of the gene expression data, a network of functional associations, and the occurrence and detection of *cis*-acting regulatory motifs in bicluster upstream sequences. Biclustering resulted in 300 biclusters covering 1,775 genes. An additional 159 genes, which exhibited significant change relative to the common reference across the data set, were determined by cMonkey to have unique expression patterns and were thus not included in biclusters; these 159 genes were inferred individually.

**Figure 1:**
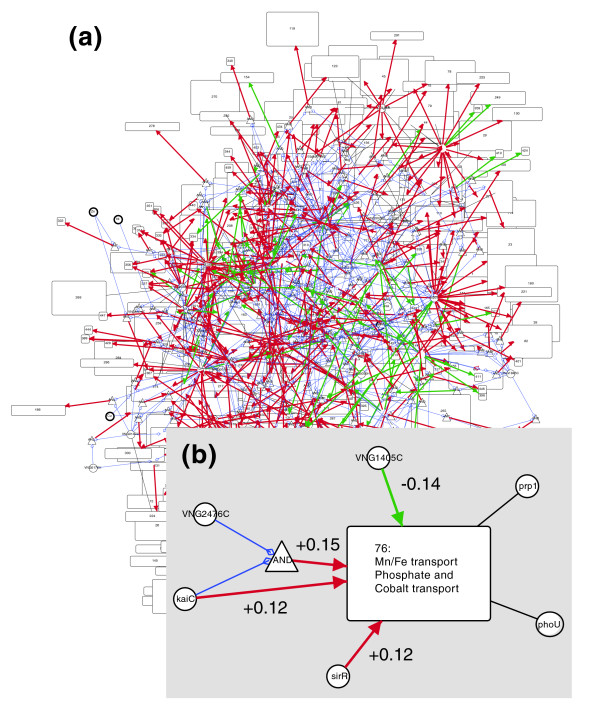
The inferred regulatory network of *Halobacterium NRC-1*, visualized using Cytoscape and Gaggle. **(a) **The full inferred regulatory network. Regulators are indicated as circles, with black undirected edges to biclusters (rectangles) that they are members of. Green and red arrows represent repression (*β *< 0) and activation (*β *> 0) edges, respectively. The thickness of regulation edges is proportional to the strength of the edge as determined by the Inferelator (*β *for that edge). Interactions are shown as triangles connected to regulators by blue edges. Weak influences (|*β*| < 0.1) are not shown. **(b) **Example regulation of Bicluster 76. The four transcription factors (TFs) *sirR*, *kaiC*, *VNG1405C*, and *VNG2476C *were selected by the Inferelator as the most likely regulators of the genes in bicluster 76 from the set of all (82) candidate regulators. The relative weights, *β*, by which the regulators are predicted to combine to determine the level of expression of the genes of bicluster 76, are indicated alongside each regulation edge. The TFs *VNG2476C *and *kaiC *combine in a logical AND relationship. *phoU *and *prp1 *are TFs belonging to bicluster 76.

The regulatory network inference procedure was then performed on these 300 biclusters and 159 individual genes, resulting in a network containing 1,431 regulatory influences (network edges) of varying strength. Of these regulatory influences, 495 represent interactions between two TFs or between a TF and an environmental factor. We selected the null model for 21 biclusters (no influences or only weak regulatory influences found, as described in Materials and methods, below), indicating that we are stringently excluding under-determined genes and biclusters from our network model. The ratio of data points to estimated parameters is approximately 67 (one time constant plus three regulatory influences, on average, from 268 conditions). Our data set is not complete with respect to the full physiologic and environmental repertoire for *Halobacterium NRC-1*, and several TFs have their activity modulated by unobserved factors (for example, post-translational modifications and the binding of unobserved ligands); the regulatory relations for many genes are therefore not visible, given the current data set. Figure 1 shows the resultant network for *Halobacterium NRC-1 *in Cytoscape, available as a Cytoscape/Gaggle web start [33,34].

An example of the predicted regulation of a single bicluster, bicluster 76 (containing genes involved in the transport of Fe and Mn; Table 1), is shown in Figure 1b. Among the 82 possible regulators, four were selected as the most likely regulators of this bicluster. The learned function of these TFs allows prediction of the bicluster 76 gene expression levels under novel conditions, including genetic perturbations (for example, to predict the expression levels in a *kaiC *knockout strain, the influence of kaiC can be removed from the equation by setting its weight to zero). We discuss the predicted regulatory model for bicluster 76 further below.

**Table 1:** Functional summary of bicluster 76: transport process putatively regulated by *sirR*

Gene	Name	Function
VNG0451G	*phoU*	Transcriptional regulator
VNG0452G	*pstB2*	Phosphate transport ATP-binding
VNG0453G	*pstA2*	Phosphate ABC transporter permease
VNG0455G	*pstC2*	Phosphate ABC transporter permease
VNG0457G	*phoX*	Phosphate ABC transporter periplasmic phosphate-binding
VNG0458G	*prp1*	Phosphate regulatory protein homolog
VNG0535C	*VNG0535C*	Membrane protein of Unknown Function
VNG1632G	*cbiQ*	Cobalt transport protein
VNG1634G	*cbiN*	Cobalt transport protein cbiN
VNG1635G	*cbiM*	ABC-type cobalt transport system, permease component.
VNG2093G	*glnA*	Glutamine synthetase
VNG2302G	*yuxL*	Acylaminoacyl-peptidase
VNG2358G	*appA*	Oligopeptide binding protein
VNG2359G	*appB*	Oligopeptide ABC permease
VNG2361G	*appC*	Oligopeptide transport permease protein
VNG2365G	*appF*	Oligopeptide ABC transporter ATP-binding
VNG2482G	*pstB1*	Phosphate ABC transporter ATP-binding
VNG2483G	*pstA1*	Phosphate ABC transporter permease
VNG2484G	*pstC1*	Phosphate transporter permease
VNG2486G	*yqgG*	Phosphate ABC transporter binding
VNG2529G	*dppB2*	Dipeptide ABC transporter permease
VNG2531G	*dppC1*	Dipeptide ABC transporter permease
VNG2532H	*VNG2532H*	Membrane protein of Unknown Function
VNG6262G	*zurM*	ABC transporter, permease protein
VNG6264G	*zurA*	ABC transporter, ATP-binding protein
VNG6265G	*ycdH*	Adhesion protein
VNG6277G	*ugpB*	Glycerol-3-phosphate-binding protein precursor
VNG6279G	*ugpA*	Sn-glycerol-3-phosphate transport system permease
VNG6280G	*ugpE*	Sn-glycerol-3-phosphate transport system permease
VNG6281G	*ugpC*	Sn-glycerol-3-phosphate transport system ATP-binding

**Figure 2:**
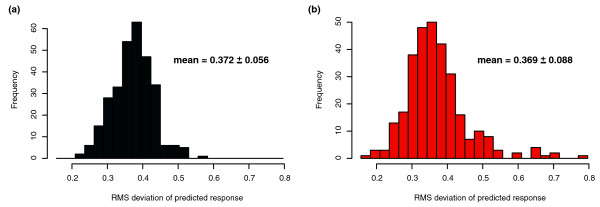
Predictive power of inferred network on biclusters. **(a) **The root mean square deviation (RMSD) error of predicted response in comparison with the true response for the 300 predicted biclusters evaluated over the 268 conditions of the training set. **(b) **The RMSD error of the same 300 biclusters evaluated on new data (24 conditions) collected after model fitting/network construction.

**Figure 3:**
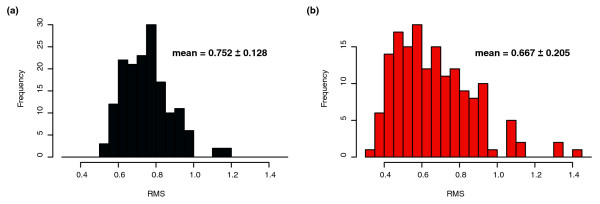
Predictive power on genes with unique expression profiles. Histograms of root mean square deviation (RMSD) of predicted response versus measured response, as calculated in Figure 2. **(a) **The RMSD error of predicted to true response for the 159 genes that cMonkey identified as having unique expression patterns and were therefore not included in any bicluster. **(b) **The same error over new data collected after model fitting/network construction for these 159 isolates.

We evaluated the ability of the inferred network model to predict the expression state of *Halobacterium NRC-1 *on a genome-wide basis. For each experimental condition, we made predictions of each bicluster state, based on the levels of regulators and environmental factors, and compared predicted expression values with the corresponding measured state (using root mean square deviation [RMSD] to evaluate the difference, or error, as described under Materials and methods, below). In this way we evaluated the predictive performance of the inferred network both on experiments in the training data set and on the 24 experiments in the independent test set (which we refer to as the newly collected data set). The expression level of a bicluster is predicted from the level of TFs and environmental factors that influence it in the network, at the prior time point (for time course conditions) or the current condition (for steady state conditions). The error estimates for the 300 biclusters and 159 single genes are shown in Figures 2 and 3. For the biclusters, the mean error of 0.37 is significantly smaller than the range of ratios observed in the data (because all biclusters were normalized to have variances of about 1.0 before model fitting), indicating that the overall global expression state is well predicted. Our predictive power on the new data (Figures 2 and 3, right panels) is similar to that on the training data (the mean RMS over the training set is within 1 standard deviation of the mean RMS over the new data), indicating that our procedure is enforcing reasonable parsimony upon the models (using L1 shrinkage coupled with tenfold cross-validation [CV], as described under Materials and methods, below) and accurately estimating the degree to which we can predict the expression levels of biclusters as a function of TF and environmental factor levels.

Although the majority of biclusters have new data RMS values well matched by the training set RMS values, there are also nine biclusters (biclusters 1, 37, 77, 82, 99, 137, 161, 165, and 180) with RMS values significantly higher in the new data than in the training data. We were unable to identify any features of these outlying biclusters (coherence of bicluster, bicluster size, variance in and out of sample for the biclusters, and so on) that distinguish them from other biclusters. We also investigated predictive performance for the 159 genes that were not included in biclusters by cMonkey. We found good predictive performance (over the new data as well as over the training data) for approximately half of these genes - a much lower success rate than seen for genes represented by biclusters. There are a number of possible explanations for this diminished ability to predict genes that also elude biclustering. Averaging expression levels over genes that are co-regulated within biclusters can be thought of as signal averaging, and thus single genes are more prone to both systematic and random error than bicluster expression levels. Another possible explanation is that these elusive genes are under the influence of TFs that interact with unobserved factors, such as metabolites. There are also about five conditions that we fail to predict well relative to the other 264 conditions (large RMS values in training and new data; Figures 2 and 3). Not surprisingly, these five conditions are all situated directly after large perturbations in time series, when the system is fluctuating dramatically as it re-establishes stasis.

We also performed several tests to determine how well our model formulation and fitting procedure performed compared with three simplified formulations, as described in detail in Additional data file 1. Briefly, these additional tests show that our current formulation for temporal modeling is essential to the performance of this procedure (mean RMSD with no temporal modeling 0.40; significance of comparison with full model *P *< 10^-10^, by paired *t* test) and produces significantly more parsimonious models. They also show that models constrained to a single predictor per bicluster perform significantly worse over the new data (mean RMSD with only a single predictor per bicluster 0.43; *P *< 10^-16^). Finally, the additional tests show that our inclusion of interactions in the current model formulation improves predictive power (mean RMSD with no interactions 0.41, *P *< 0.03).

### Homeostatic control of key biologic processes by the previously uncharacterized *trh *family

**Figure 4:**
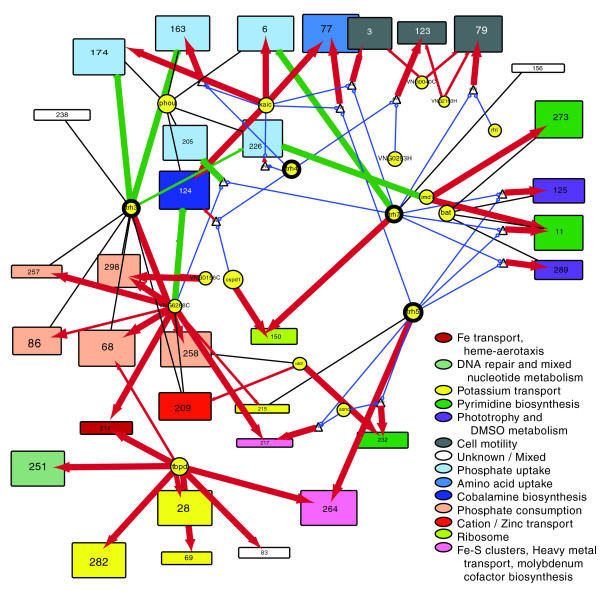
Core process regulation/homeostasis, including diverse transport process, by *trh3*, *trh4*, *trh5*, *trh7*, *tbpD*, and *kaiC*. Biclusters (rectangles with height proportional to the number of genes in the bicluster and width proportional to the number of conditions included in the bicluster) are colored by function, as indicated in the legend. In cases where multiple functions are present in a single bicluster the most highly represented functions are listed.

**Figure 5:**
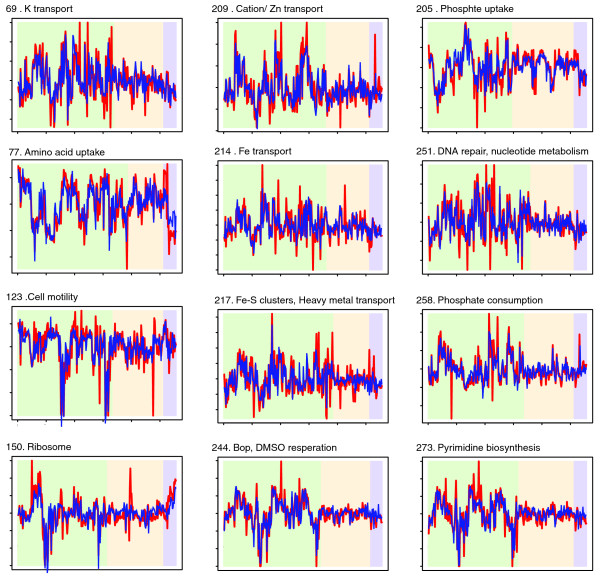
Predictive performance on biclusters representing key processes. Each plot shows a bicluster with a dominant functional theme from Figure 4. The red line indicates the measured expression profile, and the blue line shows the profile as predicted by the network model. Conditions in the left-most region of each plot were included in the bicluster, the middle regions show conditions excluded from the bicluster, and the right-most region of each plot corresponds to the 24 measurements that were not part of the original data set. The two right-most regions of each plot, therefore, demonstrate predictive power over conditions not in the training set. The estimation model parameters was done using only left-most/green conditions.

The *trh *family of regulators in *Halobacterium *(including *trh1 *to *trh7*) are members of the *LrpA*/*AsnC *family, regulators that are widely distributed across bacterial and archaeal species [35]. Their specific role in the regulation of *Halobacterium NRC-1 *genes was, before this study, unknown. We predict that four of the trh proteins play a significant role in coordinating the expression of diverse cellular processes with competing transport processes. Figure 4 shows a Cytoscape layout of the subnetwork surrounding *trh3*, *trh4*, *trh5*, and *trh7*. There is significant similarity in the functions represented by the biclusters regulated by each of the trh proteins, giving some indication that the learned influences have biologic significance. Moreover, each trh protein regulates a unique set of biclusters. Using the predicted subnetwork we can form highly directed hypotheses as to the regulation mediating the homeostatic balance of diverse functions in the cell. Our prediction for *trh3*, for example, is that it is a repressor of phosphate and amino acid uptake systems and that it is co-regulated with (and thus a possible activator of) diverse metabolic processes involving phosphate consumption. *Trh3 *thus appears to be key to *Halobacterium NRC-1 *phosphate homeostasis (a limiting factor in the *Halobacterium *natural environment). Similar statements/hypotheses can be extracted from the learned network for other regulators of previously unknown function; in this way, the network represents a first step toward completing the annotation of the regulatory component of the proteome. Figure 5 shows the predicted expression profile for 12 of the biclusters shown in Figure 4.

### Experimental verification of regulatory influences

We now briefly describe three cases in which predicted regulatory influences were supported by further experimentation.

#### *VNG1179C *activates a Cu-transporting P1-type ATPase

We predict that bicluster 254, containing a putative Cu-transporting P1-type ATPase, is regulated by a group of correlated TFs containing *VNG1179C *and *VNG6193H *- two regulators with putative metal-binding domains [28]. These regulators made attractive targets for further investigation. The Inferelator predicts that *VNG1179C *and/or *VNG6193H *are transcriptional activators of *yvgX *(a member of bicluster 254). *VNG1179C *is a *Lrp*/*AsnC *family regulator that also contains a metal-binding TRASH domain [35,36]. Strains with in-frame single gene deletions of both *VNG1179C *and *yvgX *(one of the proposed targets and known copper transporter) resulted in similar diminished growth in presence of Cu. Furthermore, recent microarray analysis confirmed that, unlike in the wild-type, *yvgX *transcript levels are not upregulated by Cu in the *VNG1179C *deleted strain. This lack of activation of *yvgX *in the *VNG1179C *deletion strain resulted in poor growth in presence of Cu for strains with a deletion in each of the two genes (Kaur A, Pan M, Meislin M, El-Geweley R, Baliga NS, personal communication).

#### *SirR *regulates key transport processes

**Figure 6:**
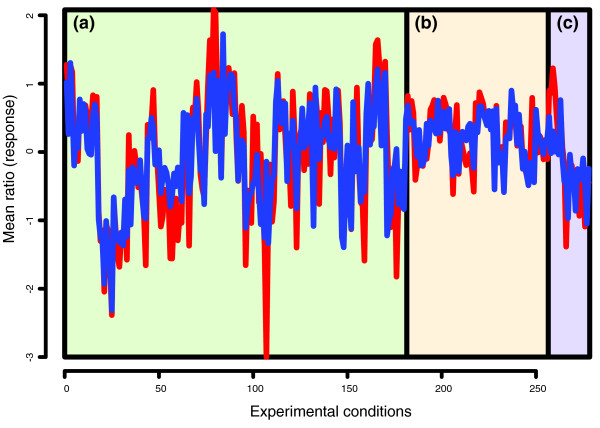
Measured and predicted response for transport processes (bicluster 76). Red shows the measured response of bicluster 76 over 277 conditions (mRNA expression levels measured as described under Materials and methods, in the text). Bicluster 76 represents transport processes controlled by the regulators *KaiC *and *SirR *(Figure 1b). Blue shows the value predicted by the regulator influence network. Conditions in **(a) **correspond to conditions included in bicluster 76 (conditions for which these genes have high variance and are coherent). **(b) **Shows conditions out of the bicluster but in the original/training data set. (These regions were not used to fit the model for bicluster 76, because models were fit only over bicluster conditions.) **(c) **Contains conditions/measurements that were not part of the original data set and thus were not present when the biclustering and subsequent network inference/model fitting procedures were carried out. Regions B and C demonstrate out of sample predictive power.

*SirR *was previously described as a regulator involved in resistance to iron starvation in *Staphylococcus epidermidis *and *Staphylococcus aureus*. *SirR *is possibly a Mn and Fe dependent transcriptional regulator in several microbial systems and a homolog to *dtxR *[37]. There is a strong homolog of *S. epidermidis sirR *in the *Halobacterium *genome but the role of this protein in the *Halobacterium *regulatory circuit has not been determined. We predicted that *sirR *and *kaiC *are central regulators, involved in regulation of biclusters associated with Mn/Fe transport, such as bicluster 76 (Figure 1b). Included in this bicluster are three genes, namely *zurA*, *zurM *and *ycdH*, that together encode a putative Mn/Fe-specific ABC transporter, consistent with the recent observation that *sirR *is needed for survival of metal-induced stress (Kaur A, Pan M, Meislin M, El-Geweley R, Baliga NS, personal communication). Figure 6 shows the predicted and measured expression levels for bicluster 76 as a function of inferred regulators (*sirR*, *kaiC*) for all conditions, including time series, equilibrium measurements, knockouts, and new data. Note that regulatory influences for this bicluster were inferred only using the 189 conditions (out of 268 total possible) that *cMonkey *included in this bicluster; excluded conditions were either low-variance or did not exhibit coherent expression for the genes in this bicluster. *SirR *mRNA profiles over all 268 original experimental conditions are positively correlated with transcript level changes in these three genes. However, upon deleting *SirR*, mRNA levels of these three genes increased in the presence of Mn, suggesting that *SirR *functions as a repressor in the presence of Mn, in apparent contrast to our prediction. In fact, a dual role in regulation has been observed for at least one protein in the family of regulators to which *SirR *belongs, which functions as an activator and repressor under low and high Mn conditions, respectively [38]. Although further investigation is needed, The Inferelator successfully identified part of this regulatory relationship and the correct pairing of regulator and target.

#### *TfbF *activates the protein component of the ribosome

*Halobacterium NRC-1 *has multiple copies of key components of its general transcription machinery (*TfbA *to *TfbG *and *TbpA *to *TbpF*). Ongoing studies are directed at determining the degree to which these multiple copies of the general TFs are responsible for differential regulation of cellular processes (Facciotti MT, Bonneau R, Reiss D, Vuthoori M, Pan M, Kaur A, Schmidt A, Whitehead K, Shannon P, Dannahoe S, personal communication), [39]. We predict that *TfbF *is an activator of ribosomal protein encoding genes. The ribosomal protein encoding genes are distributed in seven biclusters; all seven are predicted to be controlled by *TfbF*. This prediction was verified by measuring protein-DNA interactions for *TfbF *by ChIP-chip analysis as part of a systems wide study of *Tfb *and *Tbp *binding patterns throughout the genome (Facciotti MT, Bonneau R, Reiss D, Vuthoori M, Pan M, Kaur A, Schmidt A, Whitehead K, Shannon P, Dannahoe S, personal communication).

## Conclusion

We have presented a system for inferring regulatory influences on a global scale from an integration of gene annotation and expression data. The approach shows promising results for the Halophilic archaeon *Halobacterium NRC-1*. Many novel gene regulatory relationships are predicted (a total of 1,431 pair-wise regulatory interactions), and in instances where a comparison can be made the inferred regulatory interactions fit well with the results of further experimentation and what was known about this organism before this study. The inferred network is predictive of dynamical and equilibrium global transcriptional regulation, and our estimate of prediction error by CV is sound; this predictive power was verified using 24 new microarray experiments.

The algorithm generates what can be loosely referred to as a 'first approximation' to a gene regulatory network. The results of this method should not be interpreted as the definitive regulatory network but rather as a network that suggests (possibly indirect) regulatory interactions [27]. The predicted network model is consistent with the data in such a way that it is predictive of steady-state mRNA levels and time series dynamics, and it is therefore valuable for further experimental design and system modeling. However, the method presented, using currently available data sets, is unable to resolve all regulatory relationships. Our explicit use of time and interactions between TFs helps to resolve causality (for example, it resolves the directionality of activation edges), but tolerance to noise, irregular sampling, and under-sampling is difficult to assess at this point. Using cMonkey as a preliminary step to determine co-regulated groups also helps us to resolve the causal symmetry between co-expressed genes by including motif detection in the clustering process (for example, activators that are not self-regulating will ideally be removed from any biclusters they activate because they lack a common regulatory motif with their target genes, allowing the Inferelator to infer correctly the regulatory relationship). This assumption breaks down when activators are self-activating and correctly included in biclusters that they regulate [40]. Indeed, several TFs are found in biclusters; these TFs are denoted in our network as 'possible regulators' of biclusters that they are members of (undirected black edges in all figures) but they are not dealt with further. For example, *bat *is a know auto-regulator and is found in a bicluster with genes that it is known to regulate. In general, the current method will perform poorly in similar cases of auto-regulation because it is not capable of resolving such cases, and neither is the data set used in this work appropriate for resolving such cases.

Although this method is clearly a valuable first step, only by carrying out several tightly integrated cycles of experimental design and model refinement can we hope to determine accurately a comprehensive global regulatory network for even the smallest organisms. Knockouts and over-expression studies, which measure the dependence of a gene's expression value on genetically perturbed factors, are valuable in verifying causal dependencies. Another important future area of research will be the inclusion of ChIP-chip data (or other direct measurements of TF-promoter binding) in the model selection process [41]. Straightforward modifications to the current model selection process will allow the use of such data within this framework. For example we are currently planning ChIP-chip experiments to verify the regulatory influences of kaiC, sirR, the trh family of TFs, and several other key TFs that were predicted using this algorithm.

In the present study we opted not to investigate the predictive performance of our method on simulated data. RNA and protein expression data sets have complex error structures, including convolutions of systematic and random errors, the estimation of which is nontrivial. Real-world data sets are also far from ideal with respect to sampling (for example, the *Halobacterium *data set contains time series with sampling rates that range from one sample per minute to one every four hours). Instead, we evaluated our prediction error using CV. We have not discussed the topology (higher order structure or local motifs) of the derived network [42-44]. This was done primarily to limit the scope of the discussion.

A limitation of the present study is that we have inferred the expression of genes as a function of TF mRNA expression and measurable environmental factors. Accurate protein-level measurements of TFs will invariably have a more direct influence on the mRNA levels of the genes they regulate. Our method can be straightforwardly adapted to infer gene/bicluster mRNA levels as a function of TF protein levels, or activities, should large-scale collections of such data become available. Global measurements of metabolites and other ligands are also easily included as potential predictors given this framework (via interactions with TFs). We expect such data sets to be available soon [45] for several organisms as part of ongoing functional genomics efforts, and we can foresee no major methodologic barriers to the use of such data in the framework described here.

## Materials and methods

### Model formulation

We assume that the expression level of a gene, or the mean expression level of a group of co-regulated genes *y*, is influenced by the level of *N *other factors in the system: *X *= (*x_1_*, *x_2 _*... *x_N_*). In principle, an influencing factor can be of virtually any type (for example, an external environmental factor, a small molecule, an enzyme, or a post-translationally modified protein). We consider factors for which we have measured levels under a wide range of conditions; in this work we use TF transcript levels and the levels of external stimuli as predictors and gene and bicluster trancript levels as the response. Methods for selecting which of these factors are the most likely regulators, among all possible regulatory influence factors, are described below.

The relation between *y *and *X *is given by the kinetic equation:

Here, *Z *= (*z_1_[X]*, *z_2_[X] *... *z_P_[X]*) is a set of functions of the regulatory factors *X*. The coefficients *βj*, for {*j *= 1,2,...,*P*}, describe the influence of each element of *Z*, with positive coefficients corresponding to inducers of transcription and negative coefficients to transcriptional repressors. The choice *z*_*j*_*(X) *= *x*_*j *_for (*j *= 1, 2 ... *P *= *N*} amounts to the simple weighted linear combination of influencing factors *β*•*Z *= Σ*β*_*j*_*x*_*j *_[46]. To accommodate combinatorial logic for transcriptional control, we shall use a more general form for the function *Z *(described below). The constant *τ *is the time constant of the level *y *in the absence of external determinants.

Various functional forms can be adopted for the function *g*, called the 'nonlinearity' or 'activation' function for artificial neural networks, and the 'link' function in statistical modeling. The function *g *often takes the form of a sigmoidal, or logistic, activation function:

This form has been used successfully in models of developmental biology [47]. In this work we employ a truncated linear form for *g*:

Both Equations 2 and 3 allow for computationally efficient determination of *β *and are compatible with L1 shrinkage (described below). In this study we use Equation 3 because it allows for simultaneous determination of *β *at several values of the shrinkage parameter (LARS) [48]. Previous studies suggest that the distinction between these two forms is inconsequential given the expected error in the data [12,13].

The simplified kinetic description of Equation 1 encompasses essential elements to describe gene transcription, such as control by specific transcriptional activators (or repressors), activation kinetics, and transcript decay, while at the same time facilitating access to computationally efficient methods for searching among a combinatorially large number of possible regulators. To better understand specific details of regulation, it will almost certainly be required to follow up on specific regulatory hypotheses using more mechanistically detailed descriptions.

### Fitting of model parameters

The experimental conditions (individual global gene expression measurements within the data set used in this study) are classified either as belonging to a steady-state experiment or a time series experiment. In some cases, we refer to conditions as 'equilibrium' or 'steady-state' measurements out of convenience, but cannot know whether the system, in any strict sense, is at equilibrium; we imply only that an attempt was made to allow the system to reach equilibrium and that we have no knowledge of prior time-points within the same study. By a suitable reformulation of the kinetic equation (Equation 1) for each of these two data classes, we can combine both types of measurements into a single expression to fit the model parameters *β *and *τ*.

In a steady-state, *dy/dt *= 0 and Equation 1 reduces to the following:

*y *= *g*(*β*•*Z_SS_*)     (4)

where *Z*_*ss *_is the measured value of *Z *in the steady state. For time series measurements, taken at times (*t_1_*,*t_2 _*... *t_T_*), Equation 1 may be approximated as follows:

where *Δt*_*m*_= *t*_*m*+*1 *_- *t*_*m *_is the time interval between consecutive measurements, and *y*_*m *_and *z*_*mj *_are, respectively, the measured value of *y *and *z*_*j *_at time *t*_*m*_. In this formulation, we place no requirements on the regularity of *Δ*t_m_, and can readily use data with differing time intervals between measurements. It is important to note, however, that if sampling is performed at intervals that are longer than the time scales at which specific regulatory interactions act, those regulatory interactions will be missed in the data sampling and, correspondingly, by the model inference method. In most cases, we have little prior information on the regulation time scale, and hence use Equation 5 for all conditions that were sampled during a part of a time course. A possible limitation is that the inference procedure may misinterpret or miss entirely a regulatory interaction that actually occurs at a faster time scale. Under the stimuli we have considered, steady state is reached by six hours post-stimulation, and samples collected in that time range are therefore analyzed using Equation 4. In short, this method does not lessen the need for correct experimental design, but it facilitates using data with reasonable variation in sampling structure as well as the combination of data from different experiments.

In comparing Equations 4 and 5, it can be seen that the right hand sides are identical, allowing for simultaneous model fitting using equilibrium and time series data. Taking together all steady-state measurements and time course measurements, the left hand sides of Equations 4 and 5 can be combined into a single response vector, allowing *β *to be fit with one of the many available methodologies for multivariate regression. In regression terminology, the influencing factors, *X*, are referred to as regressors or predictors, whereas the functions *Z *specify what is often referred to as the 'design matrix'.

The time constant *τ *can be determined iteratively as follows. Beginning with an initial guess for *τ*, first find the regression solution for *β *using the multivariate regression methods of L1 shrinkage (described below); second, solve for a new *τ *that minimizes the prediction error given [49] and *g*(*β Z*); and third, repeat the first two steps until convergence. If available, results from independent experiments can be used to estimate by *τ *[50], thus reducing the number of free paramaters in the model. Taken together for all response variables, the set of all *β *s and *τ*s for all biclusters (300 in this work) and genes (159 singleton genes in this work) constitute the full model for the regulatory network.

### Encoding transcription factor interactions in the design matrix

We use the design matrix *Z *to encode interactions among predictor variables. The form of *g *in Equation 2 also specifies nonlinear interactions, but binary interactions are limited to the form (*β Z*)^2^, as obtained from the Taylor expansion of *g*(*β Z*), and combinatorial logic, a useful paradigm for describing many regulatory interactions, is thus only accommodated in a limited manner. More transparent encoding and approximation of interactions can be made by allowing functions in *Z *to be either the identity function of a single variable or the minimum of two variables. For example, the inner product of the design matrix and linear coefficients for two predictors that are participating in an interaction is:

*β***Z **= *β*_1_*x*_1 _+ *β*_2_*x*_2 _+ *β*_3 _min(*x*_1_,*x*_2_)     (6)

**Figure 7:**
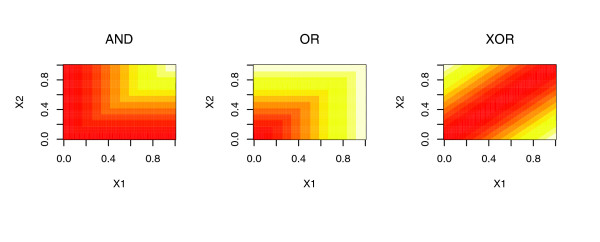
Graphical depiction of three possible interactions between predictor terms *X1 *and *X2 *(AND, OR, and XOR) that can be encoded by the design matrix *Z*. Values of *β· Z *range from 0 (red) to 1 (white). The interactions are encoded by specific linear combinations of *X1*, *X2 *and min(*X1*, *X2*), using the coefficients (three elements in the vector *β*) for the individual components (Table 2).

Using this encoding, for example, if *x*_1 _and *x*_2 _represent the levels of components forming an obligate dimer that activates *y *(*x*_1 _AND *x*_2 _required for expression of *y*), we would expect to fit the model such that *β*_1 _= 0, *β*_2 _= 0, *β*_3 _= 1. This encoding results in a linear interpolation of (linearly smoothed approximation to) the desired Boolean function. This and other interactions (OR, XOR, and AND; Figure 7 and Table 2), as well as interactions involving more than two components, are easily fit by this encoding. With this scheme for encoding interactions in the design matrix, we expect to capture many of the interactions between predictors necessary for modeling realistic regulatory networks, in a readily interpretable form. For this study we limited the procedure to binary interactions because it is unlikely that the quantity of data used would support learning beyond these pair-wise interactions. Many other methods for capturing TFs cooperatively exist as well [51].

**Table 2:** Coefficients *β *corresponding to the panels in Figure 7

	AND	OR	XOR
min(*X1*,*X2*)	1	-1	-2
*X1*	0	1	1
*X2*	0	1	1

In the table below the coefficients *β *are given, corresponding to each of the panels in Figure 7. In practice, the parameters *β *obtained from the fitting procedure can give these or any other linear combinations.

### Model selection with L1 shrinkage

**Figure 8:**
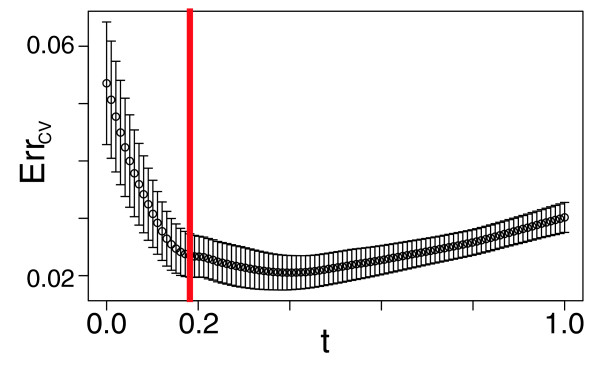
Selection of model for bicluster using cross-validation (CV). The ordinate represents an estimate of prediction error (Err_CV_) from tenfold CV (the mean of the error in the 10 leave-out samples used is the CV error estimate). The shrinkage parameter *t *allows us to select subsets of predictors continuously. We evaluate our fitted model for a range of values of *t *(with *t *= 0 [the null model] and *t *= 1 [the ordinary least squares solution]). The error bars denote the standard error of Err_CV _(the standard deviation of the 10 leave-out samples' error estimates). The red line shows the value of *t *selected for our final model for this cluster - the most parsimonious model within 1 standard error of the minimum on the Err_CV _versus *t *curve.

Given our model formulation, numerous methods have been developed for selecting subsets of predictors among candidate predictors and for estimating model parameters [52]. Including all predictors, for example, amounts to ordinary least squares multivariate regression, regressing *y *on Z. This model often has limited value in terms of interpretation and in this case would severely overfit the data. Here, we adopt the L1 shrinkage or the LASSO [48,53] for predictor selection, which involves the following minimization:

Subject to the following additional constraint:

where *β*_ols _is the ordinary least squares estimate of *β*. The shrinkage parameter *t *can range from 0 to 1. The limit *t *= 0 amounts to selection of the null model (*y *= |*y*|). In the limit *t *= 1 we have the ordinary least squares estimate for *β*, (*β *= *β *_ols_). We determine the optimal value for the shrinkage parameter by minimizing the prediction error (as estimated by tenfold CV), as shown in Figure 8. We use tenfold CV to estimate the prediction error for values of the shrinkage parameter ranging from 0 (the null model) to 1 (the ordinary least squares limit). For each value of the shrinkage parameter, this results in an error estimate (the mean on the error estimated over each of the 10 leave-out conditions; the line in Figure 8) and the standard deviation of the 10 individual leave-out error estimates (the error bars in Figure 8). We select the smallest value of *t *that is 1 standard deviation from the minimum on the CV error curve, resulting in a fairly conservative/parsimonious estimate of the shrinkage parameter [52]. In this way we select a separate value of the shrinkage parameter for each bicluster or gene we attempt to select a model for.

In this manner, we fit a predictive function for each gene and bicluster in our set resulting in a predictive, dynamic function for each gene/bicluster for which the method did not select the null model. All data used in this procedure are normalized before network inference to have row variances of 1. Thus, for a given influence on a given bicluster we can uniformly interpret the magnitude of *β*, and use the magnitude of *β *to rank the individual interactions by significance (Figure 1). We previously compared this method (L1 shrinkage with CV to select the shrinkage parameter) with several other model selection methods in the context of regulatory network inference and found it to be attractive for inferring large networks [27,54].

### Selection and search algorithms

We use a simple search strategy for fitting models for each bicluster. We exhaustively evaluate all single and pair-wise interactions saving the top five single influences and the top two pair-wise interactions. These predictors are then pooled, and L1 shrinkage is used to select the final model. Highly correlated predictors (having a Pearson correlation coefficient greater than 0.85) are pre-grouped before this search, because they are unresolvable by any data-driven method. The calculation takes less than 1 day on a single top of the line workstation (3 GHz AMD opteron). The calculation can easily be parallelized, which we have done (using PVM) and then runs in less than 1 hour on a modest cluster.

#### Algorithm outline

Given a set of biclusters, the following algorithm may be applied:

For each (bicluster k) {

   For each (TF or environmental factor *i*){

      update list of best single influences

      foreach (TF *j*){

         update list of best interactions list (min [*i*, *j*])}

      }

   Select from predictors and estimate model parameters with L1-shrinkage/CV

   Store model for gene/bicluster *k*}

Combine models for individual biclusters into global network

Process network for viewing in Gaggle/Cytoscape

### Predictive accuracy and significance testing

True signal was compared with predicted signal using RMSD:

where *Y *is the true response and  is the predicted response, *n *is the index over *N *conditions. This measure has the advantage that it does not emphasize low variance segments of the signal although several other measures of goodness are equally appropriate.

### Identification of transcription factors and putative transcription factors

A list of TFs and putative TFs was compiled using several methods including PSI-BLAST, Pfam, and COGnitor, as previously described [28].

### Microarray gene expression data set

The total data set, described in full elsewhere ('Kaur A, Pan M, Meislin M, El-Geweley R, Baliga NS' and 'Whitehead K, Kish A, Pan M, Kaur A, King N, Hohmann L, Diruggiero J, Baliga NS', personal communications), [30,31], contains genome-wide measurements of mRNA expression by microarrays in 292 conditions. The conditions included ten diverse environmental stimuli (light, Fe, Cu, Co, ultraviolet, *γ *radiation, media change, among others), gene knockouts, and samples taken at a range of times after stimulation and at steady state. By dual hybridization, each was compared with a reference condition that was identical in all 292 experiments, ensuring a high level of continuity throughout this large expression data set. The microarray slide is comprised of unique 70mer oligonucleotides spanning the 2,400 nonredundant genes encoded in the *Halobacterium *sp. *NRC-1 *genome. For each experimental condition, 8-16 replicates were assayed (including technical replicates and biologic replicates). Replicates include a reversal in the dyes (dye flip) used for labeling the RNA populations to minimize bias in dye incorporation. The significance of change was estimated by a maximum likelihood method [32], and subsequent filtering of genes with significant change was performed such that genes with fewer than five conditions with *λ *values greater than 15 were removed and set aside as genes for which we observed no significant change (where *λ *values > 15 correspond to false-positive rates of less than about 5%, based on control experiments). Of the 292 conditions, 24 had not been collected at the time that the model was fit and were thus not used in the training set. These 24 conditions were used as independent verification of the predictive power of the learned network model.

### Availability

The regulatory network and all data types used in the inference process can be visualized using the data integration and exploration tools Gaggle and Cytoscape, and can be accessed via a Cytoscape java web-start [33]. Alternate data formats are available upon request. Gaggle [55] and Cytoscape [56] are freely available on the web. Inferelator was written using the R statistical programming language [57] and is freely available upon request.

## Additional data files

The following additional data are included with the online version of this article: A Word document describing additional tests of individual components of our model formulation (Additional data file 1).

## Authors' contributions

R.B. conceived and initiated the project, developed and implemented the method and the resultant computer program, and wrote the manuscript. V.T. conceived and initiated the project, developed and implemented the method and the resultant computer program, and wrote the manuscript. D.J.R. assisted in development and implementation, and assisted with writing of the manuscript. P.S. assisted with visualization of the data/networks via the Gaggle. N.B. conceived and initiated the project, provided feedback on the quality of results, and initiated verification of results with further experimentation. M.F. performed experimental verification. L.H. provided guidance at project inception and assisted in writing the manuscript.

## Supplementary Material

Additional File 1 Additional tests of individual components of our model formulation. Click here for file
