# Supplementary material for: The Inferelator: an algorithm for learning parsimonious regulatory networks from systems-biology data sets de novo
Source: Genome Biol. 2006 May 10;7(5):R36. doi: 10.1186/gb-2006-7-5-r36 (PMC1779511; doi:10.1186/gb-2006-7-5-r36)
Supplement: Additional File 1 — Additional tests of individual components of our model formulation. [file gb-2006-7-5-r36-S1.doc]

**Supplement: Additional tests of model formulation.**

The following results show three additional tests of our current model formulation using both our training set (cross validated in-sample error) and the newly collected dataset. By disabling or simplifying parts of the model and comparing these simplified formulations to the full model we show our current formulation, and more specifically the complexity of our full model as described in the main text, to be justified. In all cases we report p-values associated with the differences between the predictive power of the simplified models (no-time, no-interaction and single-predictor) and the full model, we also report difference in predictive power as mean RMS values.

**I. Testing the significance of temporal modeling.**

Although it would be hard to argue that temporal modeling of some kind is not required to learn causation in complex systems such as free standing organisms, we acknowledge that the particular functional form we adopt or faults in the data (e.g. incorrect sampling rate or structure) could cause our temporal modeling to yield misleading results. In order to address the importance of our temporal modeing we have compared the full model (with temporal modeling, as described in the main text) to a model resulting from an identical procedure with temporal modeling disabled (using the equilibrium or steady-state data with the functional form shown in the methods section). All other parts of the procedure were identical including our encoding of interactions, the use of the Lasso to enforce parsimony and tenfold cross validation to estimate prediction error.

We find that the models produced by the full method (with temporal modeling enabled) were simultaneously more parsimonious and more predictive (produced lower prediction errors over training and new data sets).

**Table I.a Temporal modeling results in more parsimonious models: Mean number of interactions (predictors) per bicluster in the model described in the main text (Full model) as well as the model derived with no temporal modeling (No-time). Standard deviations for these mean values are also shown. Column 1 denotes the threshold value for an interaction to be counted. The trend (that the no-time model is more complex) increases as we restrict our counting of predictors to stronger predictors (absolute values of beta > 0.05 and 0.1). Column four shows the p-values for the difference between these 2 model’s complexities (for full model and no-time model) calculated over the set of all biclusters with the paired t-test and the Kolmogorov-Smirnov test (with the hypothesis set to No-time > full).**

| Predictor strength | Full model | No-time | p-value |
| --- | --- | --- | --- |
| β > 0.0 (all edges) | 4.9±1.6 | 5.4±1.6 | Paired-t: p = 4.8e-6  KS test: p = 3.9e-5 |
| β > 0.05 | 3.1±1.2 | 4.1±1.3 | Paired-t: p < 2.2e-16  KS test: p = 1.7e-15 |
| β > 0.10 | 1.8±0.87 | 2.9±1.2 | Paired-t: p < 2.2e-16  KS test: p < 2.2e-16 |

**Table I.b Temporal modeling increases predictive performance: RMS values for full and no-time models over training and validation (new) data sets. Mean RMS values are shown over both datasets as well as the estimated significance of the increase in the RMS error of the no-time model over both data sets.**

|  | Full model | No-time | p-value |
| --- | --- | --- | --- |
| Training data | 0.37 | 0.41 | Paired-t: p < 2.2e-16 |
| New/Validation data | 0.36 | 0.395 | Paired-t: p = 1.2e-10 |

**II. Testing the significance of interaction terms.**

The biological importance of modeling the interactions between transcription factors (both with other TFs and with the environment) is clear. However, from a learning standpoint it is still necessary to test the significance of our interaction terms given: 1) the data may not, as of yet, support the additional model complexity represented by the terms representing interactions between multiple predictors and 2) our method for encoding interactions could be inappropriate. We find that interactions are significant for those biclusters that in the full model had significant interaction terms (Trivially, if the initial full procedure did not result in interaction terms/predictors for a given bicluster, than disallowing interactions in these cases will produce no change in the model for that bicluster). Approximately one half of the biclusters had interaction terms as predictors in the full model. We compared the RMS values over these biclusters allowing the full model, to the RMS error of the model resulting from a run where interactions were disallowed. All RMS values are reported over the validation/new data set; we see similar trends in RMS values over the training set (*e.g.* the no-interaction RMS for a threshold of 0.05 increases to 0.42). We find that including interactions as potential predictors does indeed yield a significant improvement in predictive power, given our current data set sizes, functional form, and computational procedure.

**Table II.a: Disallowing interactions increases predictive power.** Column one shows the threshold for an interaction term to be considered in the comparison (a bicluster must have one or more interaction terms with magnitude beta greater than this value to be considered in this comparison). This ensures that the effect of removing interaction terms is measured over only those biclusters (~1/2) that had significant interaction terms in the full model; this smaller sample size does, however, decrease the significance of the interactions (higher p-values below for similar mean RMS difference). This data shows the trend that: disallowing interactions was more detrimental for biclusters that had interaction terms as significant predictors.

| Predictor strength | Full model | No-interactions | p-value |
| --- | --- | --- | --- |
| β_int_ > 0.05 | 0.36 | 0.41 | Paired-t: p = 0.030 |
| β_int_ > 0.1 | 0.37 | 0.43 | Paired-t: p = 3.1e-4 |

**III. Comparison of models selected via the full procedure to optimal single predictor models.**

We compare the full model to a heavily constrained model where for each bicluster no more than one predictor is allowed. We compare over biclusters that in the original full model had greater that a single predictor (biclusters with one or fewer predictors with beta greater than 0.05 were not considered as this would amount to comparing identical models).

**Table III.a:** For biclusters with greater than one predictor in the original mode we show RMS values over the validation data set and the p-value calculated for the decrease in predictive power upon constraining model complexity to a single predictor.

| Predictors in original model | RMS, single predictor model | Paired t-test |
| --- | --- | --- |
| >1 | 0.43 | p < 2.2e-16 |
| >2 | 0.44 | p < 2.2e-16 |
| >3 | 0.47 | p < 2.2e-16 |
| >4 | 0.47 | p < 2.2e-16 |
